# The Effects of Alcohol Hangover on Response Inhibition and Attentional Bias towards Alcohol-Related Stimuli

**DOI:** 10.3390/healthcare9040373

**Published:** 2021-03-28

**Authors:** Craig Gunn, Graeme Fairchild, Joris C. Verster, Sally Adams

**Affiliations:** 1 Addiction and Mental Health Group, Department of Psychology, University of Bath, Somerset BA2 7AY, UK; s.adams@bath.ac.uk; 2 Department of Psychology, University of Bath, Somerset BA2 7AY, UK; G.Fairchild@bath.ac.uk; 3 Division of Pharmacology, Utrecht University, 3584CG Utrecht, The Netherlands; J.C.Verster@uu.nl; 4 Centre for Human Psychopharmacology, Swinburne University, Melbourne, VIC 3122, Australia

**Keywords:** alcohol, hangover, cognition, attentional bias, mood, response inhibition

## Abstract

Alcohol hangover is associated with the development of alcohol use disorders, yet few studies have examined the influence of hangover on cognitive processes that may contribute towards future alcohol consumption such as response inhibition and attentional bias towards alcohol-related stimuli. Therefore, the current study aimed to explore the effects of hangover on these processes. In total, 37 adult drinkers who reported regularly engaging in heavy episodic drinking and experiencing a hangover at least once in the previous month took part in this within-subjects, “naturalistic” crossover study. Participants completed Go/No-Go (assessing response inhibition) and Visual Dot Probe (attentional bias) tasks in a hangover condition (morning following alcohol consumption) and a no-hangover condition (no alcohol consumption for at least 24 h). Participants also completed measures of hangover severity, mood, and perceived mental effort. Results indicated impaired response inhibition during hangover compared to the no-hangover condition (*p* < 0.001, *d* = 0.89), but no difference in attentional bias scores between conditions. Participants reported expending greater mental effort to complete tasks (*p* < 0.001, *d* = 1.65), decreased alertness (*p* < 0.001, *d* = 3.19), and reduced feelings of tranquillity (*p* < 0.001, *d* = 1.49) in the hangover versus no-hangover condition. Together, these findings suggest that alcohol hangover is associated with impaired response inhibition and lower mood. However, problems with recording eye-tracking data on the Visual Dot Probe task used in the present study may limit the reliability of our attentional bias findings.

## 1. Introduction

The most commonly reported negative consequence following a night of heavy alcohol consumption is alcohol hangover [1]. Alcohol hangover is defined as a “combination of negative mental and physical symptoms which can be experienced after a single episode of alcohol consumption, starting when blood alcohol concentration (BAC) approaches zero” [2]. Alcohol-related absenteeism, which includes hangover, costs the UK economy £1.9 billion per annum, yet its impact on productivity is still unknown [3]. Hangover may also increase the risk of developing alcohol use disorders (AUDs). The development of AUDs has been linked to hangover frequency even when controlling for previous drinking behaviour, indicating that hangover uniquely contributes towards problem drinking [4,5,6]. However, the mechanisms underlying the association between alcohol hangover and the development of AUDs are not well understood. One hypothesis is that alcohol hangover leads to impairments in response inhibition and attentional biases towards alcohol-related stimuli which jointly promote further alcohol use.

Enhanced salience of alcohol and alcohol-related stimuli, combined with reduced inhibitory control, has been theorised to contribute towards the development of AUDs [7,8,9]. Studies of acute intoxication indicate that following an alcohol prime, alcohol-related stimuli gain strong motivational properties and receive preferential attentional processing (i.e., attentional bias) [10,11]. In turn, attentional bias contributes towards alcohol-seeking behaviours and in the long-term to the development of AUDs [12,13,14]. However, attentional bias towards alcohol-related stimuli may also differ according to drinking status, with heavy social drinkers exhibiting an enhanced attentional bias towards alcohol-related cues compared with light drinkers [15,16,17,18,19,20]. Similarly, reduced response inhibition is also observed in acute intoxication [21,22] and appears to contribute towards poor decision making in recently detoxified alcohol-dependent individuals [23]. Greater impairment in inhibitory control whilst intoxicated is also associated with increased ad libitum alcohol consumption when sober [24], highlighting that poorer inhibitory abilities may contribute towards future alcohol consumption. Furthermore, alcohol-induced impairments in inhibitory control are enhanced when alcohol-related stimuli are used as targets [23,25,26]. Alongside enhanced salience of alcohol and alcohol-related cues and poorer inhibition, models of AUDs highlight that negative affect can increase alcohol-seeking behaviours via negative reinforcement [27]. Together, these studies suggest that poor response inhibition, enhanced attentional biases towards alcohol-related stimuli, and negative affect are risk factors for problematic alcohol use. Although previous studies have reported an increase in negative affect during hangover [28], few studies have examined response inhibition and attentional biases during hangover. 

Therefore, it is not known whether hangover influences attentional bias towards alcohol-related stimuli. One possibility based on anecdotal evidence (e.g., “I’ll never drink again”) is that alcohol-related cues—being related to the substance that caused the discomfort—are viewed as aversive during hangover. Animal studies have also lent support to the notion that consuming alcohol is aversive during a hangover. Gauvin et al. [29] trained rats to drink alcohol freely before injecting them with a high dose of alcohol. Consumption of alcohol decreased during the hangover stage, suggestive of avoidance. However, social drinkers mention using alcohol as a treatment for hangover, which may be effective to some extent as an individual returns to an intoxicated state [30]. In addition, avoidance of alcohol during hangover may be influenced by drinking status. For example, one study reported that 25% of students who experience hangovers have attempted to use alcohol to “cure” their hangover, and this behaviour was associated with heavier alcohol consumption in the future [31]. Furthermore, those who used alcohol to relieve hangover symptoms were more likely to meet diagnostic criteria for an AUD. Thus, the extent to which an individual avoids alcohol-related stimuli during a hangover may be related to their drinking status (i.e., heavy versus light social drinkers).

Previous studies have utilised tasks of interference control—another form of inhibition [32,33,34,35,36]—and one recent study investigated the effects of hangover on response inhibition [37]. Three naturalistic studies asked participants to complete the Eriksen Flanker and Stroop tasks, measuring different aspects of interference control, in two conditions: the morning after a night of naturalistic drinking (hangover), and again after no alcohol consumption (no-hangover) [33,34]. In one study, student participants who were experiencing a hangover exhibited greater impairments in interference control on both tasks compared to the no-hangover condition [33], whilst another found impairments on the Stroop task only [32]. In contrast, a naturalistic study that recruited participants from the general population showed no hangover effects on interference control on either task [34]. One study that experimentally induced hangover also found no evidence of hangover-related effects on interference control using the Eriksen Flanker task [35]. Furthermore, a recent study that experimentally induced hangover found a slight impairment in response selection, but not response inhibition when completing a Simon No-Go task [37]. Thus, there are currently mixed results and a need for greater clarity regarding the effects of hangover on inhibitory processes. In particular, there is a need to understand the effect of hangover following naturalistic drinking on inhibitory processes other than interference control (i.e., response inhibition).

The current study aimed to compare response inhibition and attentional bias towards alcohol-related stimuli, between hangover and no-hangover conditions in a within-subjects, crossover design. As a secondary aim, we investigated mood and broader subjective effects of hangover. Specifically, our hypotheses were: (1) participants will exhibit poorer response inhibition in the hangover compared to the no-hangover condition; (2) individuals will exhibit attentional avoidance towards alcohol-related stimuli in the hangover compared to the no-hangover condition; and (3) attentional avoidance towards alcohol-related stimuli in the hangover condition will correlate negatively with self-reported alcohol use (i.e., it will be stronger in lighter drinkers). Our secondary hypotheses were: (1) hangover severity will be positively related to response inhibition impairments and the degree of attentional avoidance; and (2) mood will be reduced and perceived effort will be increased in the hangover compared to the no-hangover condition. 

## 2. Materials and Methods

### 2.1. Participants

A total of 59 (30 Male, 29 Female) non-smoking, healthy volunteers, aged 18–30 years old, who experienced a hangover in the past month and consumed > 6 (female) or > 8 (male) units of alcohol on their “typical” night of heavy drinking were recruited predominantly from a student population. Thus, participants in this study were not hangover-resistant and regularly consumed the amount of alcohol likely to produce a hangover. Participants who consumed > 400 mg caffeine per day were excluded to avoid the possibility that acute caffeine withdrawal effects would confound performance on the cognitive tasks. Additionally, participants were not pregnant/breastfeeding, had normal/corrected-to-normal vision, had no current/past personal/family history of alcohol/drug dependency, and had no diagnosed sleep disorder. A total of 22 participants withdrew (21 following screening, 1 following no-hangover testing); thus, 37 participants (19 male, 18 female) completed the study. The University of Bath’s Psychology Research Ethics Committee approved this research (Ethics Code: 17-080).

### 2.2. Design

A within-subjects crossover “naturalistic” design was utilised, whereby participants were randomised to either the hangover (morning following alcohol consumption) or no-hangover condition (after at least 24 h of no alcohol consumption) first and then came back 1 week later to complete the tasks under the other condition. 

### 2.3. Measures

The Go/No-Go task was used as a measure of response inhibition [38]. Participants were presented with a 2 × 2 grid with a star in each section. Participants responded as quickly and accurately as possible to one of two targets (“Go” or “No-Go”) by pressing the spacebar for the “Go” target, or withholding a response to the “No-Go” target. “Go” and “No-Go” targets appeared 80% and 20% of the time, respectively. The task consisted of two blocks, with 20 practice trials and 160 experimental trials per block. In the first block, the letter “P” was the “Go” target and “R” was the “No-Go”. This was reversed in the second block. In each trial, targets randomly replaced one of the stars for a duration of 500 ms followed by an inter-stimulus interval of 1500 ms. The primary outcome measure was commission errors—failure to withhold a response to “No-Go” targets. 

**Figure 1:**
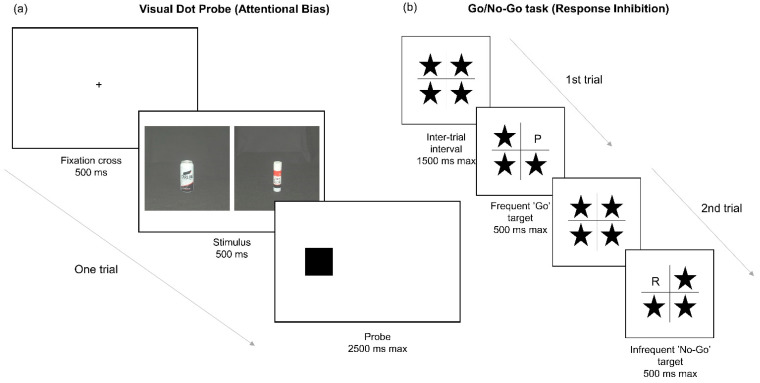
Schematic representations of the neurocognitive tasks used in this study. (**a**) Schematic representation of the Visual Dot Probe task measuring attentional bias towards alcohol-related stimuli. Participants are presented with a fixation cross followed by a pair of images, one alcohol-related (a beer can on the left in this example) and one neutral (a glue stick on the right in this example). The images are then replaced by a probe (circle or square), to which participants respond by pressing the up or down arrow on the keyboard. (**b**) Schematic representation of the Go/No-Go task used to measure response inhibition. Participants are presented with a 2 × 2 grid of stars. One of these stars is replaced by a target stimulus and participants respond by pressing the space bar for “Go” stimuli (“P”) or withholding their response to “No-Go” stimuli (“R”).

The Visual Dot Probe (VDP) task was used to measure attentional bias towards alcohol-related stimuli. We also collected eye-tracking data during the task in an attempt to improve internal validity [39]. Participants were asked to respond as quickly and accurately as possible to a visual probe (circle/square) by pressing the up or down arrows. There were 12 stimulus pairs, consisting of alcohol-related images matched on perceptual characteristics (i.e., colour and complexity) with neutral stimuli from the category “stationery”. At the beginning of each trial, a fixation cross, presented in the middle of the screen, was replaced after a stable eye fixation period of 500 ms by a stimulus pair (1 alcohol-related, 1 neutral) for 500 ms, displayed side by side. A probe replaced one of the stimuli, and participants were given 2500 ms to respond, after which there was an inter-trial interval of 500 ms. There were eight practice trials and 192 experimental trials presented in two blocks (96 trials per block). Probes replaced alcohol-related and neutral stimuli with equal frequency, and equally on each side of the screen. The 12 picture pairs appeared 16 times each, in equal frequency in each location (8 left, 8 right). Errors were removed, and reaction times (RTs) for correct responses to probes were used to calculate attentional bias scores, as per [10]. Although eye-tracking data were collected, the data were unusable due to technical error and are therefore not reported here. Figure 1 shows a schematic representation of the Go/No-Go and VDP tasks.

During screening, participants completed the Barratt Impulsivity Scale-11 (BIS-11; [40], a risk-taking questionnaire (RT-18; [41]), the trait dimension of the State-Trait Anxiety Inventory (STAI; [42]), and the Alcohol Use Disorder Identification Test (AUDIT; [43]. Estimated Blood Alcohol Concentration (eBAC) for each participants’ “typical” heavy drinking session was also calculated at screening using the Widmark formula [44].

At both testing sessions, alcohol consumption was self-reported from the previous night using pictorial prompts labelled with alcohol unit content to enable calculation of eBAC. Participants also completed the modified Alcohol Hangover Severity Scale (mAHSS), a 1-item hangover severity scale [45], the Groningen Sleep Quality Scale (GSQS; [46]), the Karolinska Sleepiness Scale (KSS; [47]), and the Alcohol Urges Questionnaire (AUQ; [48]). Participants completed mood visual analogue scales (VAS; [49]) before and after the cognitive tests and completed the rating scale of mental effort (RSME; [50]) to assess perceived mental effort during cognitive tasks.

### 2.4. Procedure

Participants attended a screening session to ensure they met the inclusion criteria, provided informed consent, and completed baseline questionnaires (BIS-11, RT-18, STAI, AUDIT). Participants were randomised to a condition order (i.e., hangover first or no-hangover first) in a within-subjects design and booked two sessions (hangover and no-hangover) according to their anticipated drinking pattern. For each participant, the time of day of testing was similar for both conditions.

On the morning of both sessions, participants were instructed to abstain from caffeine (verified by self-report) and nicotine consumption (verified by exhaled carbon monoxide < 10 ppm). As BAC levels > 0.02% can produce cognitive effects reflective of acute alcohol intoxication [51], participants were breathalysed to confirm BAC was ≤ 0.02% before testing began. Participants then completed pre-task questionnaires (KSS, GSQS, a 1-item hangover severity scale, mAHSS, AUQ, VAS) before the Go/No-Go and VDP tasks were completed in a counterbalanced order. Following the cognitive tasks, participants completed post-task measures (VAS, RSME). Upon completion of both conditions, participants were paid GBP 15 and received a full debrief.

### 2.5. Statistical Analysis

Attentional bias scores were calculated by subtracting mean RTs to probes replacing alcohol-related images from mean RTs to probes replacing neutral images, in line with [10]. Outliers were removed from data if they were > 1.5 inter-quartile range and > 2 SD from the mean. RTs < 200 or > 2000 ms were also considered as outliers and removed from the analyses [52]. Screening identified one outlier for Go/No-Go commission errors, and four outliers for VDP attentional bias scores. For VAS mood data, the 2 factors “alertness” and “tranquillity” were calculated as per [53]. The factor “alertness” comprised items such as “lethargic/energetic” and “alert/drowsy”, and the factor “tranquillity” comprised items such as “happy/sad” and “calm/excited”. We modified the statistical analysis from that specified in the pre-registered plan [54] because the data were not suitable for analysis of covariance (ANCOVA) analysis. Repeated measures analyses of variance (ANOVA) were performed using SPSS (version 24). Initially, order was included in the model as a between-subjects factor for all analyses. However, order was removed from the model if there was no evidence of it interacting with the other variables. Where data were non-normally distributed, bootstrapping of 5000 samples was performed [55].

## 3. Results

### 3.1. Participant Characteristics

Table 1 shows the participants’ demographic and clinical characteristics. Mean age of participants was 20.22 years (SD = 2.2; range = 18–28), and mean AUDIT score was 12.75 (SD = 3.96; range = 6–22). The mean number of units of alcohol consumed and eBAC reported at screening for a “typical” heavy drinking episode were 15.05 (SD = 5.41) and 0.17% (SD = 0.06; range = 0.09%–0.3%), respectively. 

### 3.2. Alcohol Consumption Prior to Hangover Condition

Participants consumed an average of 14.75 (SD = 5.64) units of alcohol, reaching an average eBAC of 0.17% (SD = 0.05) on the night before the hangover testing session. Units of alcohol consumed and eBAC did not significantly differ between the night before the hangover session and self-reported “typical” drinking occasions (*ps* ≥ 0.40), indicating that taking part in the study did not influence or change participants’ typical alcohol consumption. 

### 3.3. Effects of Hangover on Response Inhibition

**Figure 2:**
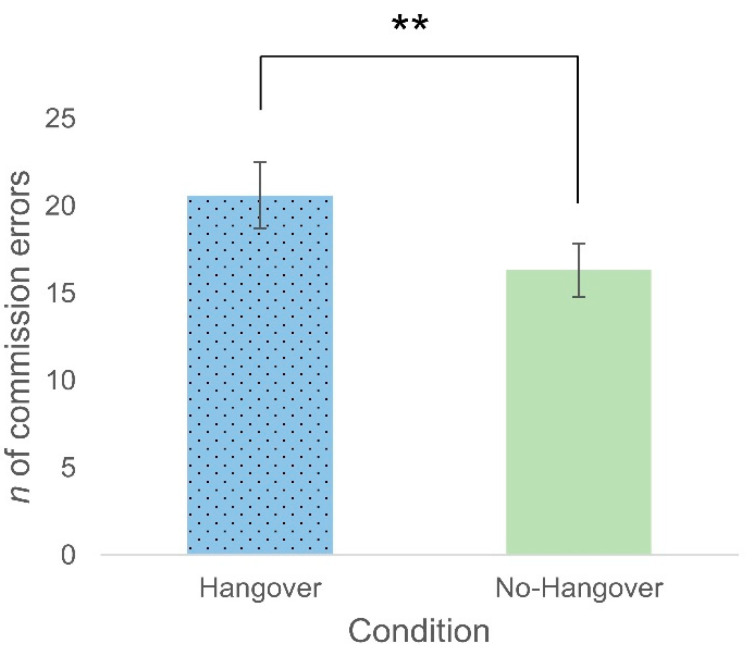
Mean number of commission errors on the Go/No-Go task in the hangover versus the no-hangover condition. A greater number of commission errors were made in the hangover condition compared to the no-hangover condition, indicating poorer response inhibition during hangover. Error bars represent ±1 standard error of the mean. ** represent a significant effect.

A paired samples t-test indicated that participants made more commission errors (*t*(35) = 3.73, *p* = 0.001, CI [2.00–6.44], *d* = 0.62) in the hangover condition (M = 20.61, SD = 11.31) than the no-hangover condition (M = 16.33, SD = 9.18; Figure 2).

### 3.4. Effects of Hangover on Attentional Bias

**Figure 3:**
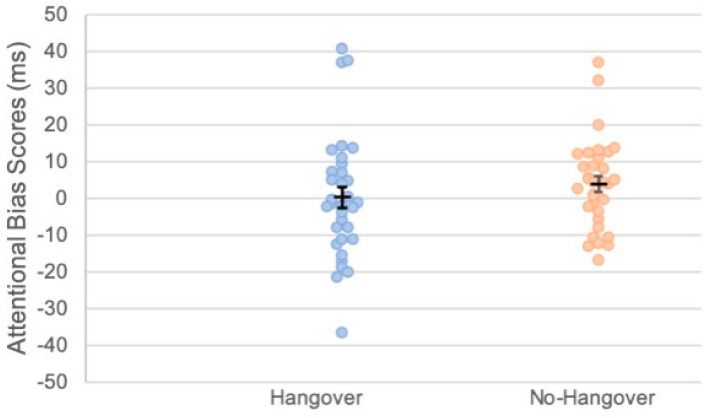
Scatter plot showing attentional bias scores towards alcohol-related stimuli for each condition. Each dot represents an individual participant’s score. Black bars indicate mean attentional bias scores. Error bars represent ±1 standard error of the mean.

Attentional bias scores were calculated in line with [10], whereby positive scores indicate attention towards, and negative scores indicate avoidance of, alcohol-related images. A significant difference between conditions would indicate that attentional biases are influenced by alcohol hangover, and the attentional bias score would indicate in which direction this occurred. A positive attentional bias score in the hangover condition relative to the no-hangover control would indicate greater attentional bias towards alcohol-related stimuli whilst hungover, whereas a negative score would indicate avoidance of alcohol-related stimuli during hangover. Mean (SD) attentional bias scores in the hangover and no-hangover conditions were 0.34 ms (2.93) and 3.96 ms (2.12), respectively. A repeated-measures ANOVA showed no main effects of condition or interactions between condition and order on attentional bias scores (*ps* > 0.31; Figure 3). Furthermore, attentional bias scores did not significantly differ from 0 in either condition (*ps* > 0.515), indicating that hangover did not influence attentional bias towards alcohol-related stimuli in either direction (avoidance or approach).

### 3.5. Correlational Analysis

Commission errors on the Go/No-Go task in the hangover condition were not correlated with alcohol consumption: AUDIT (*r* = −0.04, *p* = 0.81) or either measure of hangover severity: mAHSS (*r* = −0.08, *p* = 0.63) or 1-item hangover severity scores (*r* = −0.15, *p* = 0.36). Attentional bias scores were also not correlated with alcohol consumption (AUDIT) or hangover severity (mAHSS or 1-item hangover severity), *rs* ≤ 0.12, *ps* ≥ 0.51.

### 3.6. Subjective Measures

Repeated measure ANOVAs were conducted separately for alertness and tranquillity factors. For alertness, there was a main effect of condition (*F*(1, 33) = 83.99, *p* < 0.001, *d* = 3.19), whereby participants were less alert in the hangover condition (M = 45.49, SE = 0.67) than in the no-hangover condition (M = 54.72, SE = 0.67). This difference had a very large effect size. There was also strong evidence for a condition*time interaction (*F*(1, 33) = 12.04, *p* = 0.001, *d* = 1.21), driven by an increase in alertness scores from pre- to post-test in the hangover condition and a decrease in alertness scores from pre- to post-test in the no-hangover condition. Further, there was an order*time interaction (*F*(1, 35) = 4.29, *p* = 0.046, *d* = 0.64), which was explained by participants showing lower post-test alertness scores when they completed the no-hangover condition first. For tranquillity scores, there was a main effect of condition (*F*(1, 33) = 18.22, *p* < 0.001, *d* = 1.49), such that tranquillity was lower in the hangover (M = 44.34, SE = 0.6) than the no-hangover condition (M = 47.83, SE = 0.68). However, there was no effect of time or condition*time interaction (*ps* > 0.38). 

Separate paired *t*-tests indicated greater sleepiness (*t*(33) = 12.74, *p* < 0.001, *d* = 2.19), poorer sleep quality (*t*(34) = 8.09, *p* < 0.001, *d* = 1.37), greater perceived mental effort to complete tasks (*t*(36) = 7.09, *p* < 0.001, *d* = 1.17), and fewer urges to consume alcohol (*t*(35) = −2.39, *p* = 0.023, *d* = 0.4) in the hangover compared to the no-hangover condition (see Table 2).

## 4. Discussion

Our results suggest that participants show impaired response inhibition when experiencing a hangover, compared to when they are not hungover. Contrary to our hypothesis, there was no evidence that hangover influenced attentional bias, either in terms of avoidance or approach towards alcohol-related stimuli. Also contrary to our hypotheses, there was no relationship between attentional bias scores and levels of alcohol consumption, and no evidence that hangover severity was associated with commission errors or attentional bias scores. Secondary findings from our study revealed that participants experienced decreased alertness and tranquillity and reported that they needed to expend greater mental effort to complete the cognitive tasks when experiencing a hangover compared to the no-hangover condition.

Our results are consistent with previous naturalistic hangover studies showing poorer interference control during hangover [32,33]. Together, these findings suggest that individuals are less able to inhibit pre-potent responses during hangover. This is consistent with effects observed during acute intoxication [9], suggesting that the effects of alcohol on inhibitory control continue into the hangover stage. It is important to highlight that our findings, although consistent with other naturalistic studies of alcohol hangover, are in contrast to recent studies that experimentally induced hangover. Following administration of a set dose of alcohol to induce hangover (achieved BAC 0.11%), no evidence of hangover-related impairments in interference control was observed when completing the Eriksen Flanker task [35]. Further, another study that administered a set dose of alcohol (achieved BAC 0.13%) reported slight impairments in response selection during hangover but provided no evidence that response inhibition was influenced by hangover [37]. However, a recent systematic review highlighted that hangover-related cognitive impairments tend to be observed in studies following naturalistic alcohol consumption relative to studies that experimentally induced hangover [56]. As the effects of hangover are positively related to the amount of alcohol consumed [57], it is likely that the contrast between the results of the current study and previous experimental studies [35] may be due to higher levels of alcohol consumption by the participants in our naturalistic design.

Engaging cognitive control processes, such as inhibition, is considered effortful [58]. In the current study, participants reported expending greater mental effort in completing the cognitive tasks, including response inhibition. This increased effort may reflect a reduction in available mental resource whilst experiencing a hangover, possibly due to the processing of attentionally demanding stimuli such as painful symptoms [59], or increased fatigue [60,61,62]. As high cognitive load is known to have an impairing effect on inhibitory processes [63], the interference caused by additional processing of hangover symptoms could influence effortful cognitive processes such as inhibition. One recent study found hangover enhances the detrimental effects of cognitive load on cognitive control [35], further suggesting that cognitive resources are reduced during hangover. Together, these results suggest that hangover may adversely affect the ability to effectively engage in effortful cognitive processes (e.g., response inhibition). However, further research is required to corroborate and quantify this effect. 

In contrast to studies of acute alcohol intoxication [15,25], the current study found no evidence that hangover influences attentional bias towards alcohol-related stimuli. Contrary to our hypothesis, our results provide no support for the idea that participants will show attentional avoidance of alcohol-related stimuli in hangover. We also found no evidence for attentional biases towards alcohol-related stimuli in the hangover state. Therefore, although enhanced attentional bias towards alcohol-related stimuli may contribute towards increased alcohol-seeking behaviours during acute intoxication [9], our results suggest that these do not extend to hangover.

In line with previous research [64], secondary findings from our study showed that participants in the hangover condition experienced reduced feelings of tranquillity and perceived themselves as being less alert than in the no-hangover condition, indicating that hangover has negative subjective effects. However, although statistically significant and with a large effect size, the absolute differences in alertness were small and therefore may not be clinically or practically meaningful. 

The current findings should be interpreted in light of the following strengths and limitations. In our naturalistic design, we asked participants to engage in their “typical drinking” behaviour the evening before the hangover testing session. Our findings support the ecological validity of using a naturalistic design in hangover research, where participants’ self-reported alcohol consumption during the hangover condition did not differ from their usual drinking patterns. To resolve discrepancies between naturalistic and experimental hangover designs, future studies could consider incorporating real-time BAC tracking, which would document whether alcohol consumption is indeed higher in the former design. Controlling for individual differences in response inhibition by utilising a within-subjects design can also be considered a strength of this study. The problems with recording eye-tracking data on the Visual Dot Probe task used in the present study may limit the reliability of our attentional bias findings [65], where it is recommended that eye-tracking is used alongside behavioural measures [39]. Future studies should seek to replicate the current research with eye-tracking technology to support the current null findings for attentional biases towards alcohol-related stimuli. 

Our results add to a growing body of evidence that highlights the negative impact of alcohol hangover on cognition, which has implications for a range of stakeholders, including those who develop industrial and public policies. For example, inhibitory deficits can be associated with poor driving [66], and studies have indicated hangover can impair driving ability [67,68]. Furthermore, hangover can influence the productivity of employees in the workplace, with an estimated economic cost of GBP 2.1 billion per annum in the UK [69]. The increased mental effort to complete cognitive tasks observed in the current study may be a contributing factor to this loss of productivity. This hypothesis could be explored in future studies conducted in workplace settings.

## 5. Conclusions 

To conclude, participants exhibited poorer response inhibition during hangover versus a no-hangover control condition. Our results provide no evidence that hangover influences attentional bias towards alcohol-related stimuli (contrary to our hypothesis of attentional avoidance). Secondary findings highlight that participants report lower mood and feel that they have to expend greater mental effort to complete cognitive tasks when experiencing a hangover. Together, these findings suggest that alcohol hangover is associated with poorer response inhibition and lower mood. 

## Figures and Tables

**Table 1:** Demographic characteristics of the sample and descriptive statistics regarding their alcohol consumption.

Measures	Participants	Mean	SD
Age	Total	20.22	2.2
	Male	19.47	2.2
	Female	20.22	2.68
AUDIT	Total	12.75	3.96
	Male	13.32	3.79
	Female	12.12	4.17
‘Typical’ heavy drinking eBAC	Total	0.17	0.06
	Male	0.17	0.06
	Female	0.17	0.05
‘Typical’ heavy drinking units	Total	15.05	5.41
	Male	17.49	5.14
	Female	12.48	4.5
Previous night heavy drinking eBAC	Total	0.17	0.05
	Male	0.17	0.06
	Female	0.17	0.05
Previous night heavy drinking units	Total	14.75	5.64
	Male	17.68	5.86
	Female	11.66	3.35

*Note*. SD, Standard deviation; AUDIT, Alcohol Use Disorder Identification Test; eBAC, estimated Blood Alcohol Concentration.

**Table 2:** Descriptive statistics for the cognitive tasks and questionnaires in the hangover and no-hangover conditions.

Variable	Test	*n*	Hangover		No-Hangover		Statistic	*p*	Effect Size
			Mean	(SD)	Mean	(SD)			
Response Inhibition	Commission errors	36	20.61	(11.31)	16.33	(9.18)	*t =* 3.728	0.001	*d =* 0.62
Attentional Bias	VDP: AB scores	33	0.34	(2.93)	3.96	(2.12)	*F =* 1.054	0.312	*d* = 0.36
Hangover Severity	mAHSS	35	3.2	(1.37)	0.31	(0.32)	*t* = 13.155	<0.001	*d* = 2.22
	1-item hangover severity	35	5.51	(1.85)	0.09	(0.51)	*t* = 15.795	<0.001	*d =* 2.66
Mood	Alertness	35	45.49	(0.67)	54.72	(0.67)	*F =* 83.991	<0.001	*d* = 3.19
	Tranquility	35	44.34	(0.60)	47.83	(0.68)	*F =* 18.218	<0.001	*d* = 1.49
Mental Effort	RSME	37	76.68	(25.18)	47.55	(22.23)	*t =* 7.09	<0.001	*d* = 1.17
Alcohol Craving	AUQ	36	9.81	(3.76)	11.72	(4.81)	*t =* −2.39	0.023	*d =* 0.4
Sleep	KSS	34	6.53	(1.08)	3.56	(1.16)	*t =* 12.74	<0.001	*d = 2.19*
	GSQS	35	6.54	(2.28)	2.51	(2.2)	*t =* 8.09	<0.001	*d =* 1.37

*Note*. SD, Standard deviation; VDP, Visual Dot Probe; AB, attentional bias; mAHSS, modified Alcohol Hangover Severity Scale; RSME, rating scale of mental effort; AUQ, Alcohol Urges Questionnaire; KSS, Karolinska Sleepiness Scale; GSQS, Groningen Sleep Quality Scale.

## Data Availability

The data presented in this study are available on request from the corresponding author.
